# Soil Amendment-Mediated Herbivory Resistance, Crop Improvement, and Phytoremediation in Canola: Physiological Defense Mechanism and Health Risk Assessment

**DOI:** 10.3390/plants14071110

**Published:** 2025-04-02

**Authors:** Muhammad Wajid Javed, Mansoor ul Hasan, Muhammad Sagheer, Asim Abbasi, Mubshar Hussain, Muhammad Arshad, Dilbar Hussain, Raja Adil Sarfaraz, Razia Riaz, Nazih Y. Rebouh

**Affiliations:** 1 Ministry of Science and Technology, Islamabad 44000, Pakistan; 2 Department of Entomology, University of Agriculture, Faisalabad 38040, Pakistan; 3 Institute of Agronomy, Bahaudrin Zakaria University, Multan 60800, Pakistan; 4 Entomological Research Institute, Ayub Agriculture Research Institute, Faisalabad 38040, Pakistan; 5 Department of Chemistry, University of Agriculture, Faisalabad 38040, Pakistan; 6 Central Hitech Laboratory, University of Agriculture, Faisalabad 38040, Pakistan; 7 Department of Environmental Management, Institute of Environmental Engineering, RUDN University, 6 Miklukho-Maklaya St., Moscow 117198, Russia

**Keywords:** bio-sulfur, brassica aphid, compost, hazardous quotient, heavy metals, nutrient, phenolics, safer food consumption

## Abstract

A two-year field study was conducted using canola to check the efficacy of different soil amendment treatments (SAT), i.e., with elemental sulfur (ES), bio-sulfur (BS), and compost (Cp) mixtures against insecticide-treated (Carbosulfan) and untreated controls regarding aphid populations. The results of the experiment revealed that ES treatments significantly reduced aphid abundance, followed by Cp and ES+Cp. However, BS improved aphid herbivory. The number of siliques, seeds, thousand-seed weight, and yield were improved with a trend of ES+Cp > Cp > BS+Cp. Similarly, physiological mechanisms revealed the regulation of nutrient and phenolic contents in canola with ES improving sulfur, BS nitrogen, Cp, and ES+Cp calcium, and BS+Cp enhancing phosphorus, potassium, iron, and zinc. Furthermore, RP-HPCL indicated that ferulic acid was highest in insecticide-treated plot. Similarly, Cp improved quercetin and gallic acid; ES+Cp caffeic, chlorogenic, m-coumaric, and sinapic acid; and BS+Cp enhances syringic, vanillic, ferulic, p-coumaric, and cinnamic acid. The analysis regarding health risk assessment revealed among different SAT, ES+Cp significantly reduced the Hazardous Quotient (HQ) of Cu and Zn. However, further research is still needed to explore SAT’s potential to remediate other heavy metal stresses with possible implications for pest management in different field crops.

## 1. Introduction

Urbanization and certain other anthropogenic activities are contaminating agricultural lands on a wide scale [1]. No country in the world is totally free from such hazardous impacts of industrialization, where effluents, industrial wastes, and sewage water are affecting agricultural lands, either directly or indirectly [2]. Synthetic pesticides and chemical fertilizers are also contributing to heavy metals (HMs) contamination in soils [3]. Due to limited land resources, farmers cannot quit growing crops on such contaminated soils, especially in developing or underdeveloped economies. About 16% of land in China is contaminated with HMs, and a higher percentage is anticipated for countries like India and Pakistan [3,4]. Crops cultivated on such soils are overloaded with HMs and other harmful elements that pose serious threats to human health, particularly in children [5]. Additionally, the level of insect herbivory and their impacts on crop plants may also be affected by heavy metal contamination and effluent pollution [6].

Canola or rapeseed *Brassica napus* L. (Brassicales, Brassicaceae) is the second most important vegetable oil crop in the world [7,8]. Aphids (Aphididae: Homoptera) are a major pest of canola and other field crops at the global level [9]. They feed on leaves, inflorescence, stems, and pods of canola and cause heavy yield losses. A heavy infestation could reduce yield by up to ~75% [10]. The management of aphids relies primarily and heavily on insecticides, but they have numerous concerns and implications for the environment, specifically contaminating the water reservoirs [11], trans-generational impacts on biocontrol agents [12], and bioaccumulation of phytotoxic metals [13]. Hence, there is an urgent need to look for other environmentally friendly pest management approaches.

Soil amendment treatments (SAT) are reportedly involved in mitigating insect herbivory through the acquisition of antibiotic or antixenotic properties. Among the SAT, sulfur is considered an important component of various pesticides [7,14]; it could reduce insect herbivory by improving the defense mechanisms of crop plants [15]. It had been reported that the use of sulfur against grapevine moth (*Lobesia botrana*) reduced the survival of female moths by 43%, egg laying by 80%, emergence of neonates by 10%, and larval settlement on treated berries by 55% [16]. Similarly, among dustable and wettable formulations of sulfur, the former significantly reduced the infestation levels and oviposition preference of *Tuta absoluta* in tomato seedlings [17]. The application of elemental and bio-sulfur also hinders different fitness parameters of aphids (*Brevicoryne brassicae*) feeding on canola crop. Furthermore, sulfur treatments significantly affect the reproduction time, effective fecundity, relative growth rate, intrinsic rate of natural increase, and generation time of tested pest [7,9]. Similarly, sulfur could also enhance the quality, yield, and nitrogen utilization of crop plant [7,18]. Sienkiewicz-Cholewa and Kieloch [19] reported that the number of pods, seeds, seed weight, and seed yield per plot was higher in sulfur-treated canola than in untreated controls. Similarly, Abdallah et al. [20] showed that the application of sulfur improved seed yield, oil yield, and nitrogen-use efficiency of canola.

Compost is also an important tool in managing insect pests in different field crops, e.g., *Myzus persicae*, *Tetranychus urticae*, and *Brevicoryne brassicae* [7,18]. Vermi-compost also reduced the infestation of aphids (*B. brassicae*) on cabbage [18]. Furthermore, the use of compost also improved plant growth and per acre yield of tomato crop [21]. Several studies have reported the positive impacts of SAT on *Brassica napus*. Bharose et al. [22] recorded a significant increase in growth, seeds, and oil yield of *Brassica* spp. in sulfur-treated crop plants. Similarly, a study by Kazemeini et al. [23] also reported a higher number of pods, seeds per pod, seed weight, and plant biomass in compost-treated *B. napus*. Mixing compost with other nutrient sources improved their effectiveness for plant yield in *B. napus*. Kazemeini et al. [23] showed that the mixture of compost and urea provided the highest yield in *B. napus* over single use. Moreover, various formulations of compost have also been examined as a potential component for remedying heavy metals (HMs) [21]. Poornima et al. [3] reported the ability of composting and vermicomposting in removing HMs and other pollutants from soil. However, the role of sulfur as a potential remedy of HM stress in plants largely remains unexplored. However, Li et al. [24] reported the use of sulfur to reduce heavy metal stresses (HMSs) in edible parts of crop plants. Similarly, the production of phenolics mediated through SAT also plays a vital role in regulating certain physiological defense mechanisms in crop plants against insect herbivores [25]. They provide a secondary line of defense owing to their deterrent and toxic effects [26]. Certain flavonoids and tannins bind to proteins in insect gut and reduce nutrient absorption and ultimately impair cell growth [27]. Some phenolics, e.g., chlorogenic acid act directly against herbivores by producing reactive oxygen species [25,26,27].

Most studies have focused on sulfur nutrition for yield and quality improvement in different crops [28,29]. However, previous literature has delineated that an interrelation between SAT (sulfur, compost, and their mixture) and their phytoremediation ability is not explored in field-contaminated soils. Moreover, the core physiological mechanism involved in phytoremediation is also unclear [30,31]. To the best of our understanding, Matraszek et al. [32] have examined the role of thiol-based peptides as a vital component of sulfur-mediated remediation of HMs in plants [32]. Other mechanisms are either rudimentary or not investigated so far. In addition, the role of sulfur in the remediation of Cu and Zn has not been studied. Similarly, health risk assessment regarding consumption of Cu and Zn has not been estimated either, where canola is consumed as a regular food. Although numerous studies reported the accumulation of HMs in the roots, shoots, or leaves of canola plants, none of the studies have investigated their contents in seeds, which are the ultimate products for human consumption.

Hence, the current study was based on the hypothesis that the application of SAT could not only improve insect resistance in canola but also improve crop yield and the phytoremediation of HMs (Cu and Zn). The objective was to investigate the potential of SAT for pest resistance and phytoremediation in field environments to make canola safer for human consumption when grown in contaminated fields. A crop field was chosen from an area that had remained exposed to contaminations of effluents and sewage waste for the last 35 years. SAT were applied consecutively for two years, and their responses were compared to insecticide-treated and untreated control plots. Data for aphid abundance, crop yield attributes, nutrient and phenolic contents, daily intake (DI), and Hazardous Quotient (HQ) were assessed to generate a final outcome.

## 2. Results

### 2.1. Abundance of Aphids

**Figure 1:**
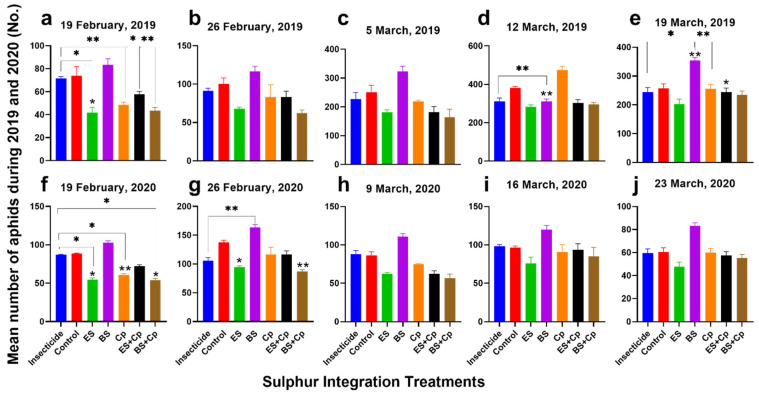
Effect of sulfur integration/soil amendment treatments (ES: elemental sulfur, BS: bio-sulfur, and Cp: compost) on the abundance of aphids (Mean ± SD; *n* = 15) on canola observed after weekly intervals in the field: (**a**) 19 February 2019; (**b**) 26 February 2019; (**c**) 5 March 2019; (**d**) 12 March 2019; (**e**) 19 March 2019; (**f**) 19 February 2020; (**g**) 26 February 2020; (**h**) 9 March 2020; (**i**) 16 March 2020; (**j**) 23 March 2020. These treatments were compared to the insecticide-treated (Carbosulfan 20% EC) and untreated control plants. Data were subjected to Repeated Measure ANOVA followed by Bonferroni test. ✱ showed statistical significance with respect to insecticide and * with respect to untreated control after Bonferroni adjustment (α = α/5 = 0.01) (*/✱: *p* < 0.01, **/✱✱: *p* < 0.001).

In 2019, cabbage aphid abundance varied significantly across treatments (F = 9.966, DF = 6, *p* = 0.0002), observation dates (F = 586.8, DF = 4, *p* < 0.0001), and their interaction (F = 38.85, DF = 24, *p* < 0.0001) (Figure 1a–e). Similar trends were observed in 2020, with significant differences among treatments (F = 32.58, DF = 6, *p* < 0.0001), dates (F = 161.8, DF = 4, *p* < 0.0001), and their interaction (F = 3.492, DF = 24, *p* < 0.0001) (Figure 1f–j).

On the first observation date (19 February 2019), ES and Cp reduced aphid abundance by 43.3% and 34.1%, respectively, compared to the untreated control (Figure 1a,f). In 2020, these treatments achieved reductions of 38.3% and 31.6%. By the second observation (26 February), reductions in 2019 were 32.3% (ES) and 17.1% (Cp), while in 2020, they were 31% and 15.5% (Figure 1b,g). In contrast, BS increased aphid numbers by 12.9% (2019) and 15.6% (2020) on the first observation, and by 16.5% (2019) and 18.7% (2020) on the second.

Combining ES or BS with Cp enhanced their effectiveness. ES+Cp reduced aphid abundance by 21.6% (2019) and 41.2% (2020), while BS+Cp achieved reductions of 41.2% (2019) and 38.9% (2020) on the first observation (Figure 1a, f). In the second observation, ES+Cp reduced aphid numbers by 16.8% (2019) and 15.2% (2020), and BS+Cp by 38% (2019) and 36.8% (2020) (Figure 1b,g).

When comparing soil amendments to insecticides on the first observation date, ES reduced aphid abundance by 41.7% in 2019 and 37.1% in 2020, while Cp achieved reductions of 32.2% and 30.3%, respectively (Figure 1a,f). Combinations like ES+Cp and BS+Cp also showed significant reductions: 19.4% and 39.5% in 2019 and 17.1% and 37.8% in 2020. However, BS had the opposite effect, increasing aphid numbers by 16.2% (2019) and 17.8% (2020). By the second observation, ES, Cp, ES+Cp, and BS+Cp continued to reduce aphid populations compared to insecticides, with BS+Cp showing the most consistent results (32.1% in 2019 and 36.8% in 2020). In contrast, BS increased aphid abundance by 26.6% (2019) and 18.7% (2020) over insecticide treatments (Figure 1b,g).

The third observation (5 March 2019 and 9 March 2020) followed a similar trend. Cp reduced aphid abundance by 12.9% (2019) and 13.1% (2020) compared to untreated controls (Figure 1c, h). ES and ES+Cp showed stronger reductions of 27.5% and 27.3% in 2019 and 27.9% and 26.7% in 2020. BS+Cp performed even better, achieving reductions of 34.3% (2019) and 33.7% (2020). However, BS alone increased aphid numbers by 28.9% (2019) and 27.6% (2020). Compared to insecticides, ES, Cp, ES+Cp, and BS+Cp all reduced aphid abundance, with BS+Cp showing the most significant reductions (33.6% in 2019 and 27.9% in 2020). Meanwhile, BS increased aphid populations by 41.7% (2019) and 36.6% (2020), highlighting its counterproductive effect (Figure 1c,h).

By the fourth observation (12 March 2019 and 16 March 2020), ES continued to reduce aphid abundance significantly, achieving reductions of 25.6% (2019) and 21.4% (2020) compared to untreated controls (Figure 1d,i). In contrast, Cp showed only minor reductions of 7.8% (2019) and 7.3% (2020). Combinations like ES+Cp and BS+Cp also demonstrated effectiveness, with reductions of 19.3% and 17.7% for ES+Cp and 19.6% and 15.5% for BS+Cp in 2019 and 2020, respectively. However, when compared to insecticide treatments, Cp and BS increased aphid numbers by 7.4% and 20.3% in 2019 and 5.6% and 19% in 2020. On the other hand, BS+Cp and ES+Cp still managed to reduce aphid abundance by 14.3% and 10.2% (BS+Cp) and 9.3% and 11.2% (ES+Cp) in 2019 and 2020, respectively, showcasing their relative effectiveness even against insecticides (Figure 1d,i).

During the final observation (19 March 2019 and 23 March 2020), ES remained the most effective treatment, reducing aphid abundance by 23.2% (2019) and 21.3% (2020) compared to untreated controls (Figure 1e,j). BS+Cp also showed modest reductions of 9.4% (2019) and 8.3% (2020). When compared to insecticides, ES outperformed all other treatments, achieving reductions of 17.3% (2019) and 20.1% (2020). Other treatments, however, were less effective, highlighting ES as the standout solution for long-term aphid control. These results emphasize the importance of treatment selection, particularly as ES consistently delivered strong results across both years.

### 2.2. Yield Attributes of Canola

**Figure 2:**
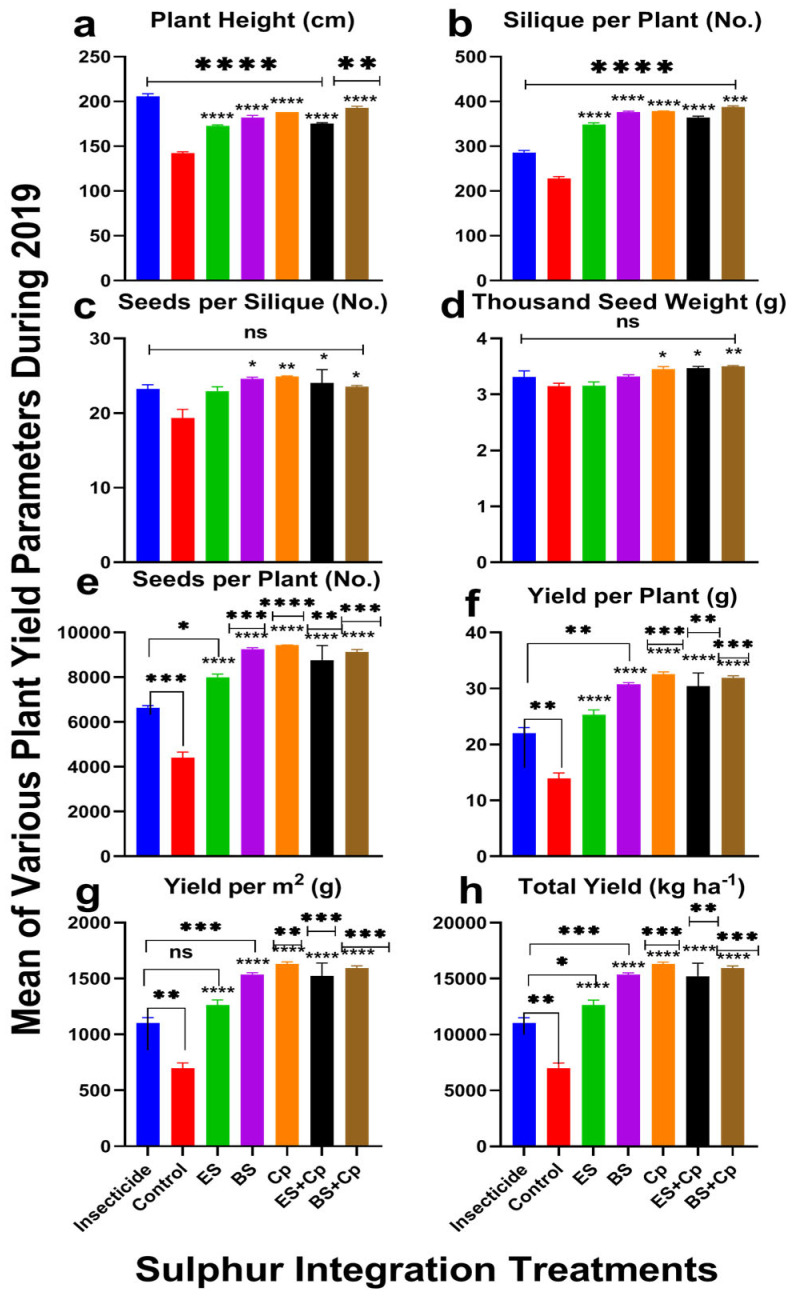
Effect of sulfur integration/soil amendment treatments (ES: elemental sulfur, BS: bio-sulfur, and Cp: compost) on yield attributes of canola in comparison to the insecticide-treated (Carbosulfan 20% EC) and untreated control plants in the field during 2019. Mean ± SD values of the following are shown: (**a**) plant height (cm); (**b**) siliques per plant (No.); (**c**) seeds per siliques (No.); (**d**) thousand-seed weight (g); (**e**) seeds per plant (No.); (**f**) yield per plant (g); (**g**) yield per m^2^ (g); (**h**) total yield (kg ha^−1^). Data were analyzed using One-Way ANOVA following Tukey multiple comparison test (*p* < 0.05). ✱ showed statistical significance with respect to insecticide and * with respect to untreated control (ns: *p* > 0.05, */✱: *p* < 0.05, **/✱✱: *p* < 0.01, ***/✱✱✱: *p* < 0.001, ****/✱✱✱✱: *p* < 0.0001).

**Figure 3:**
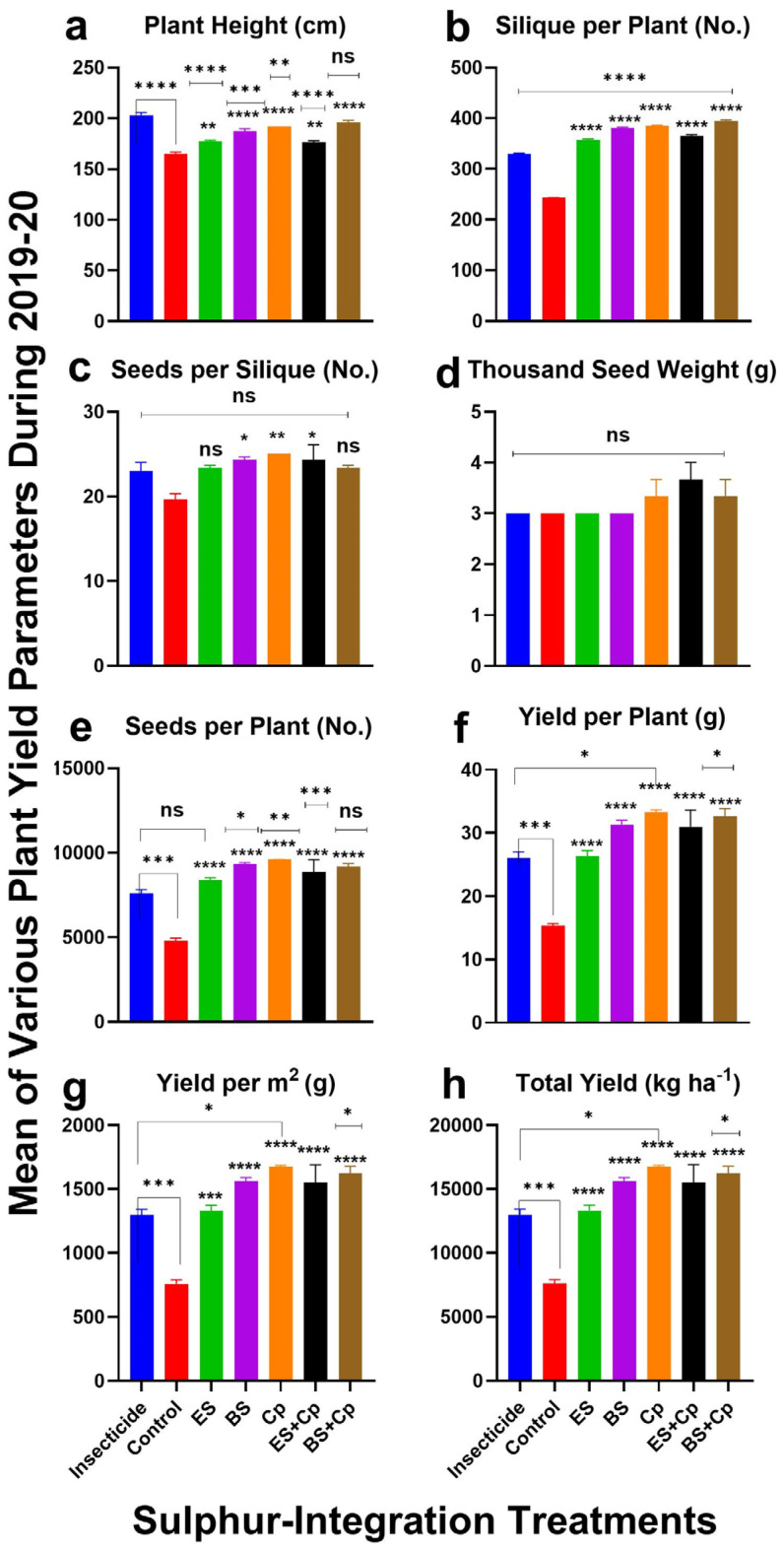
Effect of sulfur integration/soil amendment treatments (ES: elemental sulfur, BS: bio-sulfur, and Cp: compost) on yield attributes of canola in comparison to the insecticide-treated (Carbosulfan 20% EC) and untreated control plants in the field during 2020. Mean ± SD values of the following are shown: (**a**) plant height (cm); (**b**) siliques per plant (No.); (**c**) seeds per siliques (No.); (**d**) thousand-seed weight (g); (**e**) seeds per plant (No.); (**f**) yield per plant (g); (**g**) yield per m^2^ (g); (**h**) total yield (kg ha^−1^). Data were analyzed using One-Way ANOVA following Tukey multiple comparison test (*p* < 0.05). ✱ showed statistical significance with respect to insecticide and * with respect to untreated control (ns: *p* > 0.05, */✱: *p* < 0.05, **/✱✱: *p* < 0.01, ***/✱✱✱: *p* < 0.001, ****/✱✱✱✱: *p* < 0.0001).

Plant height varied significantly across treatments in both 2019 (F = 130.3, DF = 6, *p* < 0.0001) and 2020 (F = 62.64, DF = 6, *p* < 0.0001), with greater improvements observed in 2019 compared to 2020. When compared to the untreated control, ES and BS significantly increased plant height by 13.7% and 19.3% in 2019 and by 14.0% and 20.1% in 2020, respectively. Cp also performed well, boosting plant height by 22.5% (2019) and 23.8% (2020), but BS+Cp yielded even enhanced effectiveness, with increases of 25.2% (2019) and 26.4% (2020). ES+Cp showed more modest improvements, at 13.6% (2019) and 14.3% (2020). However, when compared to insecticide treatments, none of the soil amendments outperformed the insecticide, though BS+Cp and Cp came closest, showing slightly better results (Figure 2 and Figure 3a).

The number of siliques per plant varied significantly across treatments in both 2019 (F = 371.2, DF = 6, *p* < 0.0001) and 2020 (F = 927.8, DF = 6, *p* < 0.0001) (Figure 2 and Figure 3). ES increased pod numbers by 48.5% (2019) and 50% (2020), while BS boosted them by 59.2% (2019) and 61% (2020) compared to untreated controls. Cp performed even better, with increases of 61% (2019) and 62.3% (2020). Combining ES or BS with Cp further enhanced results: ES+Cp increased pods by 54% (2019) and 55.2% (2020), while BS+Cp achieved the highest improvements at 65.2% (2019) and 66% (2020). Compared to insecticides, BS+Cp showed the most significant improvement (26% in 2019 and 27.4% in 2020), followed by Cp, BS, ES+Cp, and ES (Figure 2 and Figure 3b).

Seeds per pod also differed significantly among treatments in 2019 (F = 4.622, DF = 6, *p* = 0.0086) and 2020 (F = 4.371, DF = 6, *p* = 0.0108) (Figure 2 and Figure 3c). ES increased seeds per pod by 17.3% (2019) and 18.9% (2020), while BS and Cp showed even greater improvements: 23.4% and 25.8% for BS, and 26% and 27.8% for Cp. ES+Cp and BS+Cp also performed well, with increases of 22.3% and 23.8% (ES+Cp) and 18.3% and 19.9% (BS+Cp). However, none of the treatments outperformed the insecticide in this category.

Thousand-seed weight showed significant differences among treatments in 2019 (F = 6.732, DF = 6, *p* = 0.0016) but not in 2020 (F = 1.444, DF = 6, *p* = 0.2664) (Figure 2 and Figure 3d). ES had no effect on seed weight, but BS and Cp improved it slightly: 5.9% and 10.5% (2019) and 6.3% and 11.5% (2020), respectively. Combining Cp with ES or BS provided additional improvements: 10.8% and 11.4% (2019) and 10.3% and 11.8% (2020). Compared to insecticides, only ES+Cp and BS+Cp showed slight improvements.

The number of seeds per plant varied significantly in both years (2019: F = 44.62, DF = 6, *p* < 0.0001; 2020: F = 29.43, DF = 6, *p* < 0.0001). ES, BS, and Cp increased seed numbers by 62.5%, 65.7%, and 73.4% in 2019 and by 63.3%, 67.3%, and 75% in 2020, respectively. Combining ES or BS with Cp further improved results: 69.5% and 74.7% (ES+Cp) and 71.3% and 74% (BS+Cp). Compared to insecticides, BS and Cp showed the most improvement (29.8% and 33.5% in 2019; 31.1% and 34% in 2020), while ES provided only half the benefit. Combining Cp with ES or BS further enhanced seed numbers (Figure 2 and Figure 3e).

Seed yield per plant varied significantly in both years (2019: F = 38.44, DF = 6, *p* < 0.0001; 2020: F = 25.45, DF = 6, *p* < 0.0001) (Figure 2 and Figure 3f). ES increased yield by 63.4% (2019) and 65.69% (2020), while BS and Cp achieved higher improvements: 69% and 71.5% (BS) and 75% and 78.7% (Cp). Combining ES or BS with Cp further boosted yields 76.3% and 77.8% (ES+Cp) and 77.4% and 79.6% (BS+Cp). Compared to insecticides, Cp and BS+Cp showed the most significant improvements, while ES provided only modest gains (Figure 2 and Figure 3f).

Similar results were observed for yield per m^2^ (Figure 2 and Figure 3g) and yield (Kg ha^−1^). ES significantly increased yield, while BS and Cp provided even greater improvements. Combining ES or BS with Cp did not offer additional benefits. Compared to insecticides, Cp showed the highest yield improvements (38.5% in 2019 and 39.3% in 2020), followed by BS+Cp, BS, ES+Cp, and ES.

### 2.3. Nutrient Defense Responses

**Figure 4:**
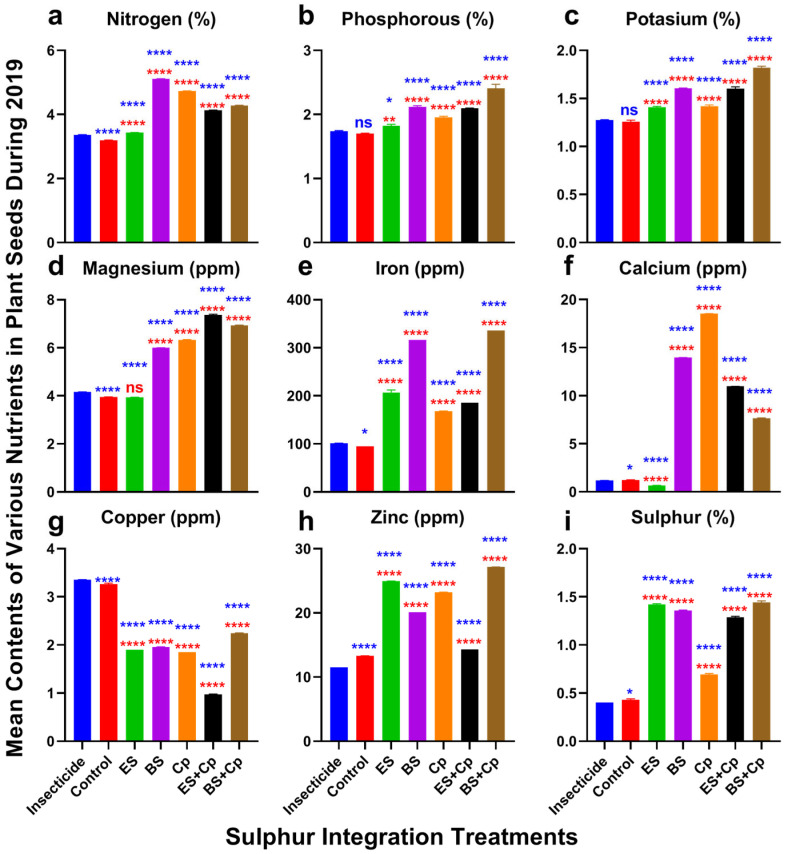
Effect of sulfur integration/soil amendment treatments (ES: elemental sulfur, BS: bio-sulfur, and Cp: compost) in comparison to the insecticide-treated (Carbosulfan 20% EC) and untreated control plants on the contents of various nutrients in the seeds of canola in the field during 2019. Bars indicated the mean contents ± SD (*n* = 5). (**a**) Nitrogen (%). (**b**) Phosphorous (%). (**c**) Potassium (%). (**d**) Magnesium (ppm). (**e**) Iron (ppm). (**f**) Calcium (ppm). (**g**) Copper (ppm). (**h**) Zinc (ppm). (**i**) Sulfur (%). Data were analyzed using One-Way ANOVA following Tukey multiple comparison test (*p* < 0.05). Blue asterisks showed statistical significance with respect to insecticide, and red asterisks with respect to untreated control (ns: *p* > 0.05, *: *p* < 0.05, **: *p* < 0.01, ****: *p* < 0.0001).

**Figure 5:**
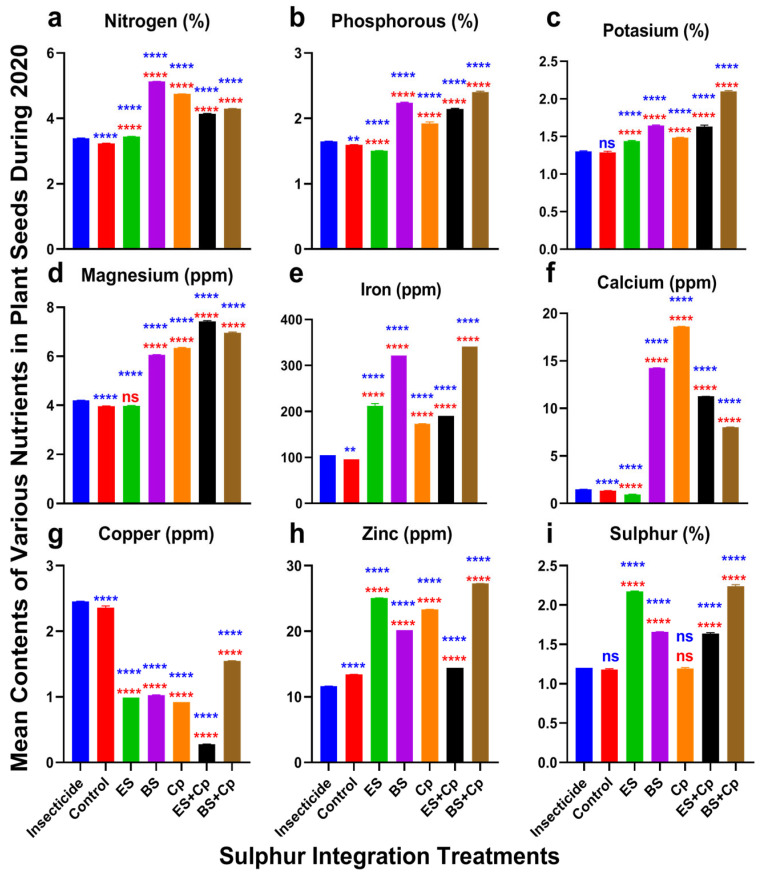
Effect of sulfur integration/soil amendment treatments (ES: elemental sulfur, BS: bio-sulfur, and Cp: compost) in comparison to the insecticide-treated (Carbosulfan 20% EC) and untreated control plants on the contents of various nutrients in the seeds of canola in the field during 2020. Bars indicated the mean contents ± SD (*n* = 5). (**a**) Nitrogen (%). (**b**) Phosphorous (%). (**c**) Potassium (%). (**d**) Magnesium (ppm). (**e**) Iron (ppm). (**f**) Calcium (ppm). (**g**) Copper (ppm). (**h**) Zinc (ppm). (**i**) Sulfur (%). Data were analyzed using One-Way ANOVA following Tukey multiple comparison test (*p* < 0.05). Blue asterisks showed statistical significance with respect to insecticide, and red asterisks with respect to untreated control (ns: *p* > 0.05, **: *p* < 0.01, ****: *p* < 0.0001).

Nitrogen content varied significantly among treatments in both 2019 (F = 34,814, DF = 6, *p* < 0.0001) and 2020 (F = 34,118, DF = 6, *p* < 0.0001) (Figure 4 and Figure 5a). ES slightly increased nitrogen levels, while Cp provided a modest additional boost compared to the insecticide and untreated control. BS, however, substantially enhanced nitrogen content. Combining ES with Cp (ES+Cp) further improved nitrogen levels over ES alone, but BS+Cp showed no additional gain compared to BS alone.

Phosphorus content also differed significantly across treatments in 2019 (F = 237.9, *p* < 0.0001) and 2020 (F = 1871, DF = 6, *p* < 0.0001) (Figure 4 and Figure 5b). Similarly, potassium content showed significant variation in 2019 (F = 691.6, DF = 6, *p* < 0.0001) and 2020 (F = 1780, DF = 6, *p* < 0.0001) (Figure 4 and Figure 5c). ES and Cp slightly improved phosphorus and potassium levels, while BS provided a substantial increase compared to the insecticide and untreated control. Combining BS with Cp (BS+Cp) yielded the best results for these nutrients.

Magnesium levels varied significantly among treatments in 2019 (F = 17,766, DF = 6, *p* < 0.0001) and 2020 (F = 17,881, DF = 6, *p* < 0.0001) (Figure 4 and Figure 5d). ES showed no improvement over the insecticide or untreated control, while BS and Cp substantially increased magnesium content. Combining sulfur formulations with Cp provided additional gains, though these were modest.

Iron content also differed significantly in 2019 (F = 6528, DF = 6, *p* < 0.0001) and 2020 (F = 6726, DF = 6, *p* < 0.0001) (Figure 4 and Figure 5e). Cp slightly increased iron levels, ES provided a small additional boost, and BS substantially enhanced iron content compared to the insecticide and untreated control. However, mixing sulfur formulations with Cp did not further improve iron levels.

Calcium content showed significant variation in 2019 (F = 48,550, DF = 6, *p* < 0.0001) and 2020 (F = 48,282, DF = 6, *p* < 0.0001) (Figure 4 and Figure 5f). ES had no effect on calcium levels, while BS and Cp substantially increased them. Combining ES with Cp (ES+Cp) improved results further, but BS+Cp showed no additional benefit over BS alone.

### 2.4. Phytoremediation of Cu and Zn

Copper also revealed significant responses among treatments in 2019 (F = 17,729, DF = 6, *p* < 0.0001) and 2020 experiments (F = 16,149, DF = 6, *p* < 0.0001) (Figure 4 and Figure 5g). All treatments reduced copper contents compared to the insecticide and untreated control. The reduction was higher in ES+Cp, followed by ES, BS, Cp, and BS+Cp over the insecticide and untreated control. Zinc contents also varied significantly among treatments in 2019 (F = 34,277, DF = 6, *p* < 0.0001) and 2020 (F = 34,269, DF = 6, *p* < 0.0001). Adding BS or Cp increased its contents slightly. Adding ES improved it more than BS and Cp, compared to the insecticide-treated and untreated plants. The mixture of BS with Cp (BS+Cp) reported the best result, while ES+Cp showed no further increase than ES (Figure 4 and Figure 5h). Sulfur contents were also improved in both formulations of sulfur and significantly differed among treatments in 2019 (F = 6496, *p* < 0.0001) and 2020 (F = 5936, *p* < 0.0001) (Figure 4 and Figure 5i). They were higher in ES and BS, followed by Cp, in comparison to the insecticide and untreated control. Mixing ES or BS into Cp gave no additional gain over their individual use.

### 2.5. Phenolic Defense Responses

**Figure 6:**
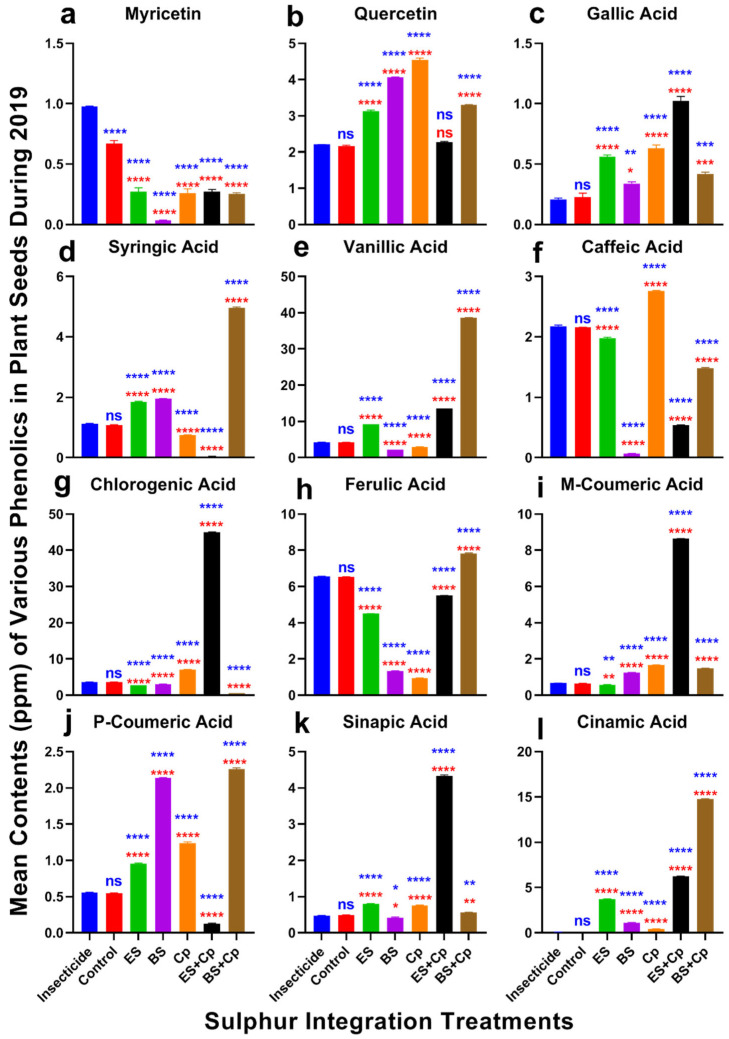
Effect of sulfur integration/soil amendment treatments (ES: elemental sulfur, BS: bio-sulfur, and Cp: compost) on the contents of various phenolics (ppm) in the seeds of canola in the field during 2019. Bars indicated the mean contents ± SD (*n* = 3). (**a**) Myricetin. (**b**) Quercetin. (**c**) Gallic Acid. (**d**) Syringic Acid. (**e**) Vanillic Acid. (**f**) Caffeic Acid. (**g**) Chlorogenic Acid. (**h**) Ferulic Acid. (**i**) M-Coumaric Acid. (**j**) P-Coumaric Acid. (**k**) Sinapic Acid. (**l**) Cinnamic Acid. Data were analyzed using One-Way ANOVA following Tukey multiple comparison test (*p* < 0.05). Blue asterisks indicated statistical significance with respect to insecticide, and red asterisks with respect to untreated control (ns: *p* > 0.05, *: *p* < 0.05, **: *p* < 0.01, ***: *p* < 0.001, ****: *p* < 0.0001).

**Figure 7:**
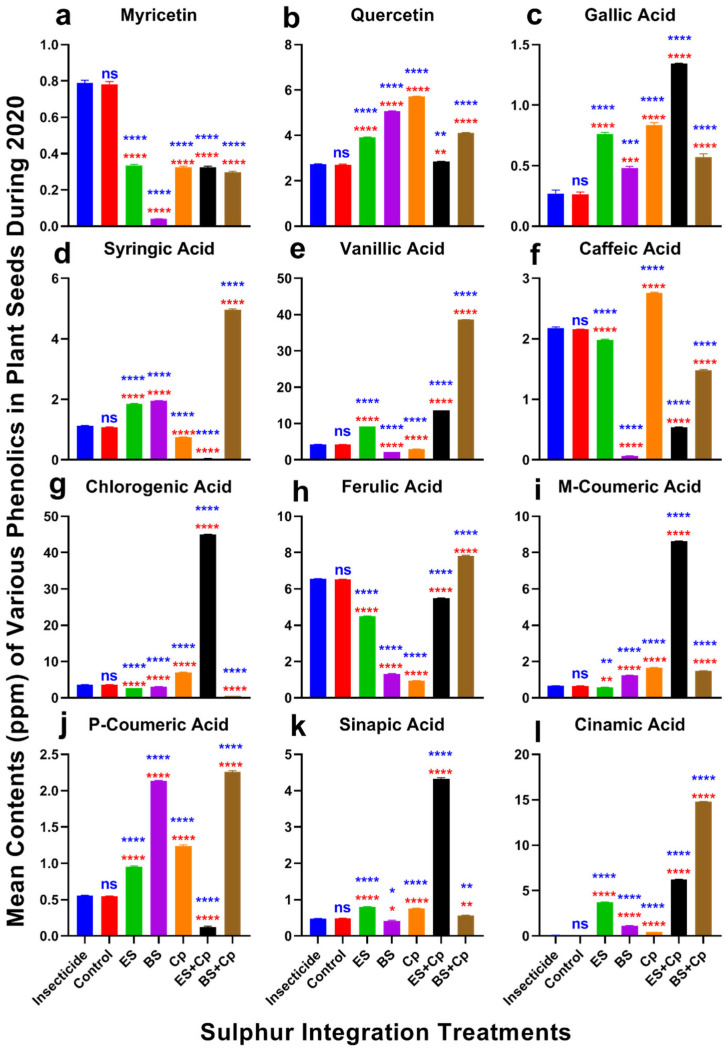
Effect of sulfur integration/soil amendment treatments (ES: elemental sulfur, BS: bio-sulfur, and Cp: compost) on the contents of various phenolics (ppm) in the seeds of Brassica napus in the field during 2020. Bars indicated the mean contents ± SD (*n* = 3). (**a**) Myricetin. (**b**) Quercetin. (**c**) Gallic Acid. (**d**) Syringic Acid. (**e**) Vanillic Acid. (**f**) Caffeic Acid. (**g**) Chlorogenic Acid. (**h**) Ferulic Acid. (**i**) M-Coumaric Acid. (**j**) P-Coumaric Acid. (**k**) Sinapic Acid. (**l**) Cinnamic Acid. Data were analyzed using One-Way ANOVA following Tukey multiple comparison test (*p* < 0.05). Blue asterisks indicated statistical significance with respect to insecticide, and red asterisks with respect to untreated control (ns: *p* > 0.05, *: *p* < 0.05, **: *p* < 0.01, ***: *p* < 0.001, ****: *p* < 0.0001).

Significant differences in myricetin content were observed among treatments in both 2019 (F = 451.1, DF = 6, *p* < 0.0001) and 2020 (F = 2098, DF = 6, *p* < 0.0001) (Figure 6 and Figure 7a). Myricetin levels were lowest in BS compared to the insecticide and untreated control. However, ES, ES+Cp, and BS+Cp significantly increased myricetin content, though levels remained lower than in the insecticide treatment, which had the highest concentration.

Quercetin content also varied significantly in 2019 (F = 2546, DF = 6, *p* < 0.0001) and 2020 (F = 7828, DF = 6, *p* < 0.0001) (Figure 6 and Figure 7b). ES, BS, and Cp significantly improved quercetin levels compared to the insecticide and untreated control, but combining Cp with ES or BS provided no additional benefit. Similarly, gallic acid content increased significantly in both years (2019: F = 286.5, *p* < 0.0001; 2020: F = 758.6, *p* < 0.0001) (Figure 6 and Figure 7c). BS showed the highest improvement, followed by ES and Cp. Combining ES with Cp (ES+Cp) yielded the highest gallic acid levels, while BS+Cp showed no additional improvement.

Syringic acid content differed significantly in 2019 (F = 25,500, DF = 6, *p* < 0.0001) and 2020 (F = 12,439, DF = 6, *p* < 0.0001) (Figure 6 and Figure 7d). ES and BS improved syringic acid levels, while Cp reduced them. BS+Cp substantially increased syringic acid, whereas ES+Cp decreased it compared to the insecticide and untreated control. Vanillic acid content also varied significantly in 2019 (F = 82,559, DF = 6, *p* < 0.0001) and 2020 (F = 140,696, DF = 6, *p* < 0.0001) (Figure 6 and Figure 7e). ES increased vanillic acid levels, with ES+Cp providing a slight additional boost and BS+Cp showing substantial improvement.

Caffeic acid content showed significant differences in 2019 (F = 5345, DF = 6, *p* < 0.0001) and 2020 (F = 12,434, DF = 6, *p* < 0.0001) (Figure 6 and Figure 7f). Cp improved caffeic acid levels, while other treatments reduced them compared to the insecticide and untreated control. Chlorogenic acid content varied significantly in both years (2019: F = 44,742, DF = 6, *p* < 0.0001; 2020: F = 34,147, DF = 6, *p* < 0.0001) (Figure 6 and Figure 7g). ES+Cp increased chlorogenic acid levels, while other sulfur treatments reduced them.

Ferulic acid content also showed significant variation in 2019 (F = 19,359, DF = 6, *p* < 0.0001) and 2020 (F = 52,727, DF = 6, *p* < 0.0001) (Figure 6 and Figure 7h). BS+Cp increased ferulic acid levels, while other treatments reduced them. M-coumaric acid content varied significantly in both years (2019: F = 42,826, DF = 6, *p* < 0.0001; 2020: F = 91,810, DF = 6, *p* < 0.0001) (Figure 6 and Figure 7i). BS and Cp increased m-coumaric acid levels, while ES slightly reduced them. Combining Cp with BS (BS+Cp) provided no additional gain, but ES+Cp showed substantial improvements.

P-coumaric acid content differed significantly in 2019 (F = 1458, DF = 6, *p* < 0.0001) and 2020 (F = 11,017, DF = 6, *p* < 0.0001) (Figure 6 and Figure 7j). BS showed the highest increase, followed by Cp and ES. ES+Cp reduced p-coumaric acid levels, while BS+Cp substantially improved them. Sinapic acid content also varied significantly in both years (2019: F = 5567, DF = 6, *p* < 0.0001; 2020: F = 24,408, DF = 6, *p* < 0.0001) (Figure 6 and Figure 7k). ES+Cp improved sinapic acid levels, while other treatments had only slight effects.

Cinnamic acid content showed significant differences in 2019 (F = 82,687, DF = 6, *p* < 0.0001) and 2020 (F = 130,506, DF = 6, *p* < 0.0001) (Figure 6 and Figure 7l). Levels were negligible in the insecticide and untreated control, but sulfur treatments significantly increased cinnamic acid, with BS+Cp showing the highest improvement, followed by ES+Cp, ES, BS, and Cp.

### 2.6. Health Risk Assessment for Humans

Health risks were assessed on the basis of DI and HQ. DI values for Cu were higher for insecticide and infested controls. However, in SAT, values were higher for BS+Cp followed by BS and ES treatment. For Zn, DI values remained higher for BS+Cp followed by ES during both years of study (Table 1). No HQ was greater than 1, which shows that canola is safer to consume in all treatments; however, SAT revealed their potential to reduce HQ values over time with the best performance being shown by ES+Cp, ES, BS, and Cp for Cu, while ES+Cp followed by BS and Cp shown best for Zn in both years (Table 2).

## 3. Discussion

### 3.1. Effect of SAT on Aphid Abundance

Sulfur is ranked as the fourth most important nutrient for plants, but only a few studies have investigated its role against insect pests. Previously, different formulations of compost were used to combat notorious field insect pests [14]. However, the novelty of this study relies on the integrated use of compost (Cp) and bio-sulfur (BS) for managing canola aphids. In the present study, the use of elemental sulfur and compost reduced the abundance of aphids; however, compost showed more promising results, which are in accordance with the findings of Mahmoud et al. [33] and Ahouangninou et al. [34], where compost demonstrated higher rates of efficacy in controlling the population of tomato leafminer (*Tuta absoluta*), whiteflies (*Bemisia tabaci*), mites (*Tetranychus urticae*), and aphids (*Myzus persicae*). Prior to an aphid attack, the crop defense mechanism would be activated by using SAT during crop sowing. Results showed that ES, followed by ES+Cp, Cp, and BS+Cp treatments, surpassed the role of insecticide and control group (non-treated). On the other hand, the sole application of BS treatment positively influences the number of aphids in comparison to insecticide and control. It is asserted that these treatments suppressed the aphid abundance for approximately 3 weeks soon after their infestation, slowly reducing their effectiveness over time. This reduction is caused by multiple factors such as quality deterioration in the host plant, intensified plant growth, and a slight rise in temperature [35]. Our study proposed that the split applications of sulfur treatments during different stages of crop development could ultimately foster pest management. Among multiple treatments, the use of ES followed by ES+Cp showed improved efficacy than others.

In the current study, treatment of both formulations of sulfur significantly reduced aphid abundance. These results are in line with the findings of Tacoli et al. [16], who reported the insecticidal efficacy of sulfur dust against grapevine moth (*Lobesia botrana*). Similarly, Zappala et al. [17] stated a noteworthy decrease in *Tuta absoluta* population after the application of sulfur to tomato plants. Different researchers also indicated the role of compost in mitigating pest infestation [33,34]. Compost may have different mechanisms to reduce pest abundance, which may involve better uptake of nutrients and activation of enzymes, e.g., chitinase, which affects molting in insects. Moreover, Mahmoud et al. [33] also reported the involvement of certain phenolic compounds in suppressing insect pests. However, further studies at molecular levels are needed to reveal its fundamental mode of action.

### 3.2. Effect of SAT on Yield Attributes

The application of either formulation of sulfur and compost significantly improved canola yield attributes. Among the treatments, BS+Cp had the highest effectiveness, followed by ES+Cp, Cp, BS, and ES, all of which outperformed conventional insecticide treatments and controls. On average, soil amendments resulted in yield improvements of 64.5% in ES, 70.3% in BS, 76.9% in Cp, 77% in ES+Cp, and 78.5% in BS+Cp compared to the untreated control. When compared to insecticide treatment, these improvements were 26.8% in ES, 30.6% in Cp, 38.9% in BS, 29.9% in ES+Cp, and 34% in BS+Cp. Soil amendments enhanced seed yield by promoting root elongation, allowing greater nutrient uptake for increased pod and seed production. Compost application, in particular, boosted seed yield by enriching the soil with organic matter, nitrogen, and essential nutrients. In BS treatments, the availability of phosphorous and potassium likely contributed to improved plant growth. Supporting this, Bharose et al. [22] observed a significant increase in growth, seed yield, and oil content in sulfur-treated *Brassica* spp. (toria), while Demir and Basalma [36] reported similar effects in sulfur-treated sunflowers.

The yield-enhancing effects of compost were attributed to improved seed germination, seedling development, and flowering, likely due to the induction of phytohormones. Kazemeini et al. [23] found that compost-treated canola exhibited the highest number of pods, seeds per pod, seed weight, and plant biomass compared to untreated plants. Compost also improved nutrient availability and soil water retention capacity [37]. Furthermore, mixing compost with other nutrient sources synergizes its efficiency in improving plant yield. Kazemeini et al. [23] reported that a compost–urea combination produced the highest canola yields compared to either used alone. However, further research is needed to establish a comprehensive nutrient management strategy that integrates compost with other fertilizers for optimal plant health and pest control.

### 3.3. Effect of SAT on Phytoremediation

Various compost formulations have been studied for their potential in remediating HMs. Asemoloye et al. [37] reported that spent mushroom compost reduced HM contamination in soil by 30 to 40%. Similarly, Irfan et al. [38] found that compost and biochar enhanced the phytoremediation of lead, cadmium, and chromium in maize plants. Sarathchandra et al. [39] also observed significant improvements in the remediation of Cu and Zn in the shoots of perennial ryegrass when compost was applied. In contrast, the role of sulfur as a potential remedy of HM stress in plants has been less extensively studied. Shi et al. [40] demonstrated that sulfur application reduced arsenic levels in wheat grains by 20%. Matraszek et al. [32] reported that sulfur nutrition decreased cadmium content in lettuce by 38%, while Li et al. [24] found a 29% reduction in cadmium accumulation in the shoots of Pakchoi following sulfur nutrient application.

### 3.4. Effect of SAT on Nutrient Defense

Soil treatments with either ES or BS enhanced nutrient content in canola, with mixed treatments (ES+Cp and BS+Cp) yielding better results, except for copper. While most soil amendments reduced copper levels, ES did not, indicating their potential phytoremediation ability. Both single and combined application of ES and BS increased nitrogen, phosphorous, and potassium levels compared to the untreated control, likely due to the presence of nitrogen and nitrogen-fixing actinomycetes in compost. Compost also contributed to higher phosphorous and potassium content [23]. Additionally, sulfur application improved phosphorus and potassium availability in oilseed crops such as safflower [41] and *Brassica* spp. [22].

Magnesium and iron levels also increased following soil amendments, with both single and mixed treatments showing improvements. Ikoyi et al. [42] demonstrated that sulfur application significantly enhanced iron and magnesium content in ryegrass compared to untreated plants. Similarly, calcium content was also higher in soil amendment treatments, with Cp followed by BS showing the highest calcium levels, while ES had the lowest. This increase was attributed to microbial activity in Cp and BS, which facilitated the release of soil-bound calcium. Sulfur application has also been shown to improve calcium content in ryegrass [42].

Copper levels decreased dramatically across all soil amendment treatments, with ES+Cp providing a substantial reduction, followed by BS, ES, and Cp. This decline may be linked to the phytoremediation effects of sulfur and compost. Ikoyi et al. [42] also reported a noticeable reduction in copper after sulfur application in ryegrass. In contrast, zinc levels were higher in sulfur and compost-treated soils, with BS+Cp and ES showing the highest concentrations. Previous studies have also shown that sulfur application enhances zinc content in safflower [41] and sunflower [36].

Sulfur content also improved in ES, BS, and Cp treatments compared to both insecticide-treated and untreated control, with single and mixed treatments performing equally well. While ES and BS naturally contained sufficient sulfur, Cp exhibited improved sulfur levels due to microbial activity. Kumar et al. [43] highlighted the role of *Arbuscular mycorrhiza* (AM) in mobilizing and enhancing sulfur uptake in plants by activating sulfur-mobilizing bacteria such as *Polaromonas, Burkholderia,* and *Azospirillum*. Overall, both sulfur formulations improved nutrient content compared to insecticide treatment and untreated controls. The combined application of sulfur and compost proved more effective than using either alone. However, further research is needed to assess the impact of soil amendments on microbial communities.

### 3.5. Effect of SAT on Phenolic Defense

Sulfur treatments (ES and BS) and their combinations with compost (Cp) enhanced various phenolic compounds in canola. Aphid feeding also stimulated the production of certain phenolics, particularly flavonols such as myricetin and quercetin. Myricetin levels increased in insecticide-treated plants as well, while the lowest content was observed in the BS treatment. In contrast, ES and mixed treatments had the highest Myricetin levels, highlighting the role of sulfur fertilization in enhancing phenolic compounds. Quercetin was most abundant in compost-treated plants, followed by those treated with bio-sulfur. Li et al. [44] reported that sulfur application increased quercetin content in mustard, while compost combined with other bio-fertilizers improved quercetin in tomato plants [21].

The ES+Cp and BS+Cp treatments resulted in higher concentrations of gallic and vanillic acid, respectively. This aligns with findings by Tian et al. [45], who reported that sulfur application enhanced gallic and vanillic acid content in winter wheat seeds. Similarly, caffeic acid was highest in compost-treated plants, emphasizing the role of compost and bio-fertilizers in increasing caffeic acid levels [46]. Ibrahim and Balah [47] highlighted compost’s ability to enhance phenolic content by conditioning the soil and stimulating the activities of soil microbiomes. Additionally, ferulic acid was most abundant in BS+Cp treatments, followed by ES+Cp. Sulfur application was also effective in increasing ferulic acid content in *Brassica rapa*, while the combination of sulfur and compost yielded superior results compared to sulfur alone in *Brassica juncea* [48].

ES+Cp treatments increased m-coumaric acid levels, whereas p-coumaric acid was highest in BS+Cp. Sinapic acid was more abundant in ES+Cp, while cinnamic acid levels were highest in BS+Cp, surpassing both insecticide-treated and control plants. Compost, being a natural source of phenolic compounds, contributed to higher hydroxy cinnamic acid in mixed treatments [47,48]. The addition of other nutrients to compost and bio-fertilizers also had a positive impact on phenolic compound synthesis. An increase in chlorogenic acid, ferulic acid, and caffeic acid was observed in *Brassica oleracea* following sulfur treatment. This effect may be attributed to sulfur’s ability to reduce the activity of phenol-oxidizing enzymes such as PPO and POD [49]. However, further research is needed to explore this mechanism in greater detail, particularly the role of soil microbes in triggering phenolic biosynthesis in canola.

Our study revealed the specificity of nutrient and phenolic-based mechanisms in canola, which contributed to increased resistance against herbivory. This was supported by a reduced aphid population under different SAT, compared to the insecticide-treated and non-treated controls. Additionally, the improvement in key yield attributes suggests that SAT induced herbivore resistance in canola, further reinforcing their agronomic benefits.

### 3.6. Effect of SAT on Human Health Risk

The HQ was below 1 in all treatments, indicating that canola grown in the studied contaminated soil was safe for consumption. SAT effectively reduced HQ values, highlighting the phytoremediation potential of sulfur and compost applications. It is common practice to assess the HQ values after washing vegetables or edible plant parts, which can result in lower values than those found in unwashed produce used in kitchens. However, in this study, the edible seeds consumed either directly or processed into edible oil were assessed. To obtain higher estimates that reflect real field conditions, soil particles were also considered as they serve as the primary source of HMs contamination, potentially influencing dietary intake and HQ values [50].

In future studies, environmental factors, e.g., the soil type, temperature, and composition of microbial communities, should be assessed as they affect SAT performance. Seasonal variations, rainfall patterns, and microbial activity should also be considered. Additionally, agronomic practices, including crop rotation, soil tillage, weeding methods, and irrigation practices, may influence the effectiveness of SAT. Long-term monitoring of heavy metal bioavailability, plant uptake patterns, and soil health indicators (e.g., organic matter, nutrient levels) is also essential. Furthermore, evaluating the interaction between SAT and local flora and fauna, as well as assessing the potential for heavy metal leaching or runoff, would provide a more comprehensive understanding.

## 4. Materials and Methods

### 4.1. Features of the Experimental Area

The present experiments were executed in the Department of Entomology, University of Agriculture, Faisalabad (UAF), Pakistan. The recorded ranges of temperature, relative humidity, and rainfall during the experimental period from November to March are given in Table 3.

### 4.2. Physical and Biochemical Properties of the Soil

Soil characteristics could have an impact on the efficiency of studied treatments. Therefore, the physical and biochemical characteristics of the soil were determined before the sowing of the crop. Soil characteristics were examined using standard procedures of the International Center for Agriculture Research in the Dry Areas (ICARDA) [51]. Briefly, soil samples were dried for 36–48 h in wooden trays (temperature 35 ± 3 °C and humidity 55 ± 5%). After that, samples were transformed into well-ground powdery material using a grinding mill and then sieved through a 2 mm sieve. The obtained soil samples were stored for further analysis.

Soil texture was estimated by determining the percentages of sand, silt, and clay in the soil. The soil texture was sandy clay loam in both years. Additionally, saturation (%), electrical conductivity (dSm^−1^), organic matter (%), calcium and magnesium ions (mmolc/L), total copper contents (mg kg^−1^), total iron contents (mg kg^−1^), total nitrogen (%), available phosphorous (ppm), extractable potassium (ppm), sulfur (ppm), and zinc (mg kg^−1^) were measured (Table 4). Properties of irrigation water were also determined during both study years with reference to total soluble salts (2018–19: 6 meq/L and 2019–20: 8 meq/L), residual sodium carbonate (1.6 meq/L in both study years), and sodium absorption ratio in (2018-19: 7 milli mole/L and 2019–20: 8 milli mole/L).

### 4.3. Sowing of Canola and Field Management Practices

*Brassica napus* cv. “Faisal Canola” was procured from Oilseed Research Institute (ORI), Faisalabad, Pakistan, and sown in a contaminated field area of 15 × 15 m^2^ (31.41865 E, 73.07798 N, elevation 184 m, Faisalabad City, Punjab, Pakistan). Land was plowed and disc harrowed before sowing during first week of November in 2018 and 2019. Seeds were sown in rows using a small seed drill at the rate of 5 kg seeds (~1 million seeds) per hectare. An average density of 50 plants per m^2^ was maintained in each sub-plot (3 × 2 m) having a row–row distance of 35–40 cm and plant-plant distance of 4–5 cm. Fertilizers were applied at recommended doses per crop cycle, i.e., 90 kg Nitrogen (Urea), 60 kg Phosphorus (P_2_O_5_) (Di Ammonium Phosphate-DAP), and 50 kg Potassium (Sulfate of Potash-SOP) per hectare. DAP and SOP were applied during the land preparation, whereas urea was divided into three split doses (i.e., during land preparation, flowering, and pod formation). In addition to this, standard agronomic procedures were followed depending on crop requirements. Plots were harvested upon recommended maturity (i.e., 30–40% plants with brown pods). After sun drying for 12–15 days (8–10% moisture), pods were threshed, and seeds were stored in brown paper bags.

### 4.4. Application of SAT in the Soil

The SAT involved soil application of elemental sulfur: ES, bio-sulfur: BS, and compost: Cp, which were applied alone and in mixtures. The efficacy of each SAT was assessed using an “Insecticide” (Carbosulfan)-treated and untreated control plots (i.e., aphid-infested plants) having no treatments. Carbosulfan (20% EC; FMC, Pakistan) was sprayed at the economic threshold level (ETL) of the targeted pest and later at an interval of 10 days at the field-recommended dose rate. The treatments were randomized in complete blocks, i.e., RCBD design, to reduce variability among field blocks. There was a separate main plot for each amendment with three sub-plots (i.e., replications). Detailed specifications of treatments are given in Table 5.

SAT were applied using the manual broadcasting method and then mixed up in soil using a conventional plow (depth 10–15 cm). Treatments of ES and BS were applied at the rate of 8000 kg ha^−1^ and Cp at the rate of 16,000 kg ha^−1^. About 4.8 kg of either ES or BS and 9.6 kg of compost was added per sub-plot (3 × 2 m^2^). The details of the treatments studied during both years are given in Table 6.

### 4.5. Abundance of Aphids in the Field

At each sampling date, five plants from each replication were chosen randomly, and the top canopy of each plant was observed to count the total number of aphids [10]. The number from each plant was considered a subsample, and the average of all five subsamples was taken, which corresponded to the number of aphids per replication.

### 4.6. Yield Attributes of Canola

Important yield attributes, i.e., plant height (cm), number of pods (siliques per plant), number of seeds per silique, thousand-seed weight (g), yield per plant (g), yield per m^2^ (g), and total yield (kg ha^−1^) were calculated. Plants were harvested from an area of one square meter from each sub-plot (replication) to estimate seed yield and other components (number of pods, seeds per pod, thousand-seed weight, and yield per m^2^). Pods were harvested manually and cut at the intersection of shoot and root. A meter rod was used to measure the length of shoots (plant height). Pods were threshed individually to count the number of seeds per pod. After that, seeds were sieved, and a representative sample was taken to measure the weight of one thousand seeds. Yield per m^2^ (g) was estimated from the harvested plants (1 m^2^ area), and then measurements were extended mathematically to estimate yield in kg ha^−1^. Yield loss in SAT-treated plots was calculated using the expression mentioned [10].

### 4.7. Nutrient Mechanism of Defense

To ensure complete variability of field, pod samples were taken from at least 10–12 fresh, fully turgid, and widely scattered plants (160–170 days old). The collected pod samples were pooled together to form one biological replicate [51]. Nutrient contents in pods were analyzed specifically from seeds. The purpose of using the seeds was to assess whether the tested treatments had impacted the seed quality and yield status [52]. The seed samples were prepared for nutrient analysis by drying (60 °C, overnight) in an oven (Memmert, Nabertherm, Germany). Seed material was ground and taken in a quantity of 0.25 g to determine the contents of nutrients using the following protocols:

#### 4.7.1. Nitrogen

Kjeldahl (digestion and distillation method) was used to determine nitrogen contents of *B. napus* seeds. Briefly, seed samples were digested in an acid (H_2_SO_4_ 98%, 10 mL) along with a catalyst mix (K_2_SO_4_-Se, 3 g). After complete digestion, sample volume was made up to 100 mL. Standard digest consisted of 0.1 g EDTA. Later on, obtained distillate was auto-titrated with acid (H_2_SO_4_ 0.01 N, pH 5.0). Nitrogen contents were calculated using mathematical expression of Estefan et al. [51].

#### 4.7.2. Phosphorus

Similarly, phosphorus contents of tested seeds were assessed using vanadate–molybdate technique [53]. Initially, the catalyst mixture of 22.5 g [(NH_4_)_6_MoO_24_.4H_2_O]/(ammonium heptamolybdate) was prepared in 400 mL of DDH_2_O. Simultaneously, ammonium metavanadate (1.25 g NH_4_VO_3_) was prepared in 300 mL heated DDH_2_O. After mixing both samples, 250 mL of HNO_3_ was added into the mix along with DDH_2_O to make a final volume of 1 L. Seed samples were digested using a few drops of pumice boiling granules and catalyst mixture (3 g), with a bit-by-bit addition of concentrated H_2_SO_4_ (10 mL). The solutions were frequently agitated on the hot plate (100 °C, Model: MSC Basic C) until the completion of digestion. The indication of complete digestion is the formation of a clear solution. After that, tubes were subjected to 380 °C temperature for about 110–120 min. The tubes were then cooled down, and the final volume was brought up to 100 mL with DI water. Each tube was divided into blank (no seed sample), standard digest (0.1 g EDTA), and internal reference (with seed sample), and absorbance values were noted at 410 nm wavelengths after 30 min.

#### 4.7.3. Potassium

Samples of dried seeds were digested using above method, and final contents were produced having 50 mL (5 mL digested seeds, and rest was diluted with DI water) using the method of Jadia and Fulekar [54]. Contents were then read on flame photometer (Sherwood 410, Sherwood Scientific Ltd., Cambridge, UK).

#### 4.7.4. Sulfur

Seed samples were assessed by the Turbidmetric method devised by LachicaáGarrido [55] with few modifications. A 3 g dried seed material was placed in the evaporating washbowl. Magnesium nitrate (15 mL) was added to spread over the sample surface. Later on, it was heated at 185 °C. Temperature was then enhanced to 285 °C to oxidize sulfur and sulfide into sulfates. The mixture was heated overnight at 440 °C in a muffle furnace (Vulcan 3-400, Neberthem, Lilienthal, Germany) until the color of the precipitate changed from brown to yellow, and white ash was left behind. The samples were then cooled and covered with watch glass. Post-moistening, concentrated HCl (15 mL) was added and then heated to boil for 2–3 min. Next, 15 mL of water was added, and watch glass was washed. The contents were then removed into the washbowl. Later on, contents of washbowl were shifted into volumetric flask (150 mL), and volume was made up to the level. After filtering the contents with the Whatman filter paper, the remaining contents were used for further assessment.

Next, 0.4–0.5 meq SO_4_–S extracts were pipetted into a graduated beaker to dilute up to 50 mL. A few drops of methyl orange along with 1 mL hydrochloric acid were added (1:1) until pink coloration. To precipitate sulfate (possibly as barium sulfate), the contents of the beaker were boiled, and BaCl_2_.2H_2_O (1 N, 10 mL) was added to it. Then, all contents were boiled for 10–12 min on the hotplate and permitted to cool. Finally, the filtrate was collected over the filter paper and washed many times with lukewarm DDH_2_O until the removal of all traces of chloride, which was confirmed using silver nitrate test.

Then, the filtrate was placed on a dry pre-weighted porcelain crucible to note the weight (Weight1) and retained to dry at 105 °C (60 min) in an oven (Memmert, GmbH+Co., Schwabach, Germany). Crucibles were transferred to muffle furnace (temperature 550 °C, Vulcan 3-400, Neberthem) for 120–180 min to dry. Later on, the crucible was removed and cooled in a desiccator to note the final weight (Weight2). Finally, sulfur contents were measured using following equation:

$$S\left(\%\right)=\frac{Weight2-Weight1}{Vs}\times \frac{1000}{0.1165}$$

where

Vs = Volume (mL) of seed extract used for measuring 0.1165 g BaSO_4_ (equal to 1 meq of SO_4_)

#### 4.7.5. Mg, Fe, Ca, Cu, and Zn

After the digestion of seed samples, Zn, Cu, and Fe were determined by atomic absorption spectroscopy (AAS Hitachi-Z-8200, Tokyo, Japan) using 50 mL diluted seed samples (having 5 mL transparent digested aliquot). Later on, calibration curves were generated, and values were measured from the expression devised by Estefan et al. [51]:

$$Zn,Fe,Cu\left(ppm\right)=ppm\;Zn,\;Fe,\;Cu(from\;calibration\;curve)\times \frac{V}{Wt}$$

where

V = Total volume of extract

Wt = Weight of dry seed samples used for digestion

However, the contents of Ca and Mg were measured by titrating the dry ash with EDTA chemical [51].

### 4.8. Phenolic Mechanism of Defense (RP-HPLC)

For estimation of phenolic compounds, 40 g of powdered seed material was extracted with 200 mL of 80% methanol using ultrasonicator (Model 50, Frequency 20 kHz, Fisherbrand, Thermo Fisher Scientific Inc., Waltham, MA, USA) (4 °C for 60 min). Afterward, the obtained mixture was centrifuged at 10,000 rpm (10 min) to isolate the supernatant and pellet. The pellet was extracted and agitated overnight at 200 rpm (4 °C) using 100 mL of extraction buffer (80% ethanol in water, acidified with 0.5% formic acid). The supernatants from previous day and present day were pooled and evaporated using rotary evaporator to get slurry. Next, the slurry was washed to attain 4 mL sample (final volume). Subsequently, the raw extractions were diluted with five volumes of methanol and filtered via Teflon membrane (0.45 µm; Sartorius Biotech, Göttingen, Germany). The obtained filtrate was then analyzed using RP-HPLC (LC-10A, Shimadzu, Kyoto, Japan) [56].

Phenolic compounds were quantified using comparison method with already established internal standards of HPLC-grade Myricetin, Quercetin, Gallic Acid, Syringic Acid, Vanillic Acid, Caffeic Acid, Chlorogenic Acid, Ferulic Acid, M-Coumaric Acid, P-Coumaric Acid, Sinapic Acid, and Cinnamic Acid obtained from Sigma-Aldrich, Darmstadt, Germany) [57]. The area obtained [mV.s] was multiplied with a correction factor to determine the final content (ppm) of a particular phenolic compound. DataApex software, Version: Clarity 7.0 (Data Handling Application for Windows OS; Prague, The Czech Republic) was used to process signals for results.

### 4.9. Health Risk Assessment for Humans

Daily intake (DI) of HM elements (Cu and Zn) was assessed based on human consumption patterns in that area, while Hazardous Quotient (HQ) was estimated as a ratio between daily intake and reference dose, which indicates the maximum safer limits of that HMs in the food. The current study included children of the age of one to five years as they are more prone to HM stress, and their feeding patterns and body weight represent higher contents of HMs in the food and body tissues as compared to the older humans [58]. DI values (mg of HMs per kg of body weight per day) were assessed on the basis of feeding patterns, body weight, and contents of HMs (i.e., heavy metals Cu and Zn) in the meal [59].

$$DI=\frac{Cf\times Ed\times Ef\times Fi\times Ir}{At\times Bw}$$

Cf = contents of HMSs in the meal (mg per kg of fresh weight)

Ed = Exposure duration to indicate how many years a human lived in that area (e.g., 5 years)

Ef = Exposure frequency to show how many times a human takes HMs containing meal per year (180 days)

Fi = is the fraction of ingested heavy metal that was absorbed in the human food system. Its value was taken as “1” because the humans in that area mostly took canola or its products as food in that area. It was assumed to ensure potential estimates on maximum levels.

Ir = Intake rate of food material (kg per meal)

At = Average exposure time in days to show number of days a human is exposed to HMs stress (e.g., 236 days)

Bw = Mean body weight of a human under study (e.g., 20 kg average)

On the other hand, the Hazardous Quotient (HQ) was also assessed. It is an indication of expected health risks against exposure to heavy metals. If HQ is less than 1, then its consumption is likely to be safe; however, a value > 1 may pose serious health concerns. HQ helps public health scientists ensure general public safety by identifying potential health substances and restricting their intake of food. HQ is estimated as follows:

HQ = DI/ORD

DI = Daily Intake of HMS food

ORD = Oral reference dose.

ORD was 0.04 for Cu and 0.3 (mg per kg per day) for Zn as per indication [59]. HQ values lesser than 1 were safer for local human populations; however, equal or higher values than 1 were unsafe/hazardous.

### 4.10. Statistical Analyses

Data were analyzed principally using GraphPad Prism Version 9.0.0 (GraphPad Software; San Diego, CA, USA). Data were initially subjected to Gaussian distribution/normality (Shapiro–Wilk) and homoscedasticity (Bartlett’s) tests to detect violations of the assumptions of ANOVA. Geisser and Greenhouse corrections were made to satisfy the additional assumptions of sphericity of repeated-measure ANOVA for aphid abundance and reproduction indices. Data violating the assumptions of ANOVA were transformed using Y = log (Y) equation or analyzed using a non-parametric test, i.e., Kruskal–Wallis test, and then subjected to Dunn’s test of multiple comparison (α = 0.05).

Data for aphid abundance were repeatedly collected from same plots; therefore, data were analyzed using repeated-measure ANOVA based on the method of [60], with time points and treatments being the two factors. Means were separated by the Bonferroni test when the results of ANOVA were significant. Bonferroni adjustment was applied to alpha values to avoid type I error (α = α/5 = 0.01). However, the effects of treatments on yield attributes of canola, nutrient contents, and phenolic compounds were analyzed using the One-Way ANOVA (and Non-Parametric Mixed) Model followed by the Tukey test (α = 0.05).

## 5. Conclusions

Soil amendments mediate resistance in canola against aphids and improve crop yield attributes and phytoremediation ability, specifically for Cu and Zn in contaminated agro-ecosystems. The population of aphids was reduced across all SAT except with BS, which had a positive effect on its population. Plant yield attributes such as plant height, number of pods, seeds per pod, thousand seed weight, and yield per plot were improved significantly. The levels of nutrient and phenolic defense chemicals were also improved. Among the nutrients, N, P, K, Ca, Mg, and S were improved exceptionally. The contents of phenolics, e.g., Myricetin, Quercetin, Gallic Acid, Syringic Acid, Vanillic Acid, Caffeic Acid, Chlorogenic Acid, Ferulic Acid, M-Coumaric Acid, P-Coumaric Acid, Sinapic Acid, and Cinnamic Acid were also improved in an SAT dependent manner, i.e., different treatments improved contents of certain phenolics. Health risk assessment indicated SAT-treated canola to be safer than insecticide or control treatments (i.e., grown on contaminated soils). However, our study was limited as it did not incorporate other heavy metals such as mercury, cadmium, chromium, etc. Moreover, similar studies should also be carried out for other vegetables. Further research is also needed to assess the ability of SAT to remediate other heavy metals with a determination of subsequent impacts on soil microbial fauna, crop yield, and food consumption.

## Figures and Tables

**Table 1:** Effect of soil amendment treatments on Daily Intake (mg of heavy metals kg^−1^ of body weight^−1^) of Copper and Zinc from plant material of canola. Data were analyzed using One-Way ANOVA following Tukey multiple comparison test (*p* < 0.05). * showed statistical significance with respect to insecticide and ^
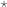
^ with respect to untreated control (ns: *p* > 0.05, *: *p* < 0.05, **: *p* < 0.01, ***: *p* < 0.001, ****: *p* < 0.0001, ^
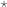
^: *p* < 0.05, ^
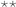
^: *p* < 0.01, ^
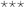
^: *p* < 0.001, ^
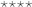
^: *p* < 0.0001).

Nature of Treatments	Field Experiments of 2019		Field Experiments of 2020	
	Copper	Zinc	Copper	Zinc
Insecticide	0.00434 ± 0.0003	0.0176 ± 0.012	0.0056 ± 0.005	0.0211 ± 0.0011
Control	0.00433 ± 0.0003 (ns)	0.0174 ± 0.012 (ns)	0.0060 ± 0.005 (ns)	0.0244 ± 0.0021 (ns)
ES	0.00331 ± 0.0002 (*,^ 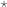 ^)	0.0407 ± 0.041 (***,^ 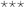 ^)	0.0022 ± 0.002 (**,^ 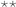 ^)	0.0460 ± 0.0051 (***,^ 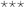 ^)
BS	0.00337 ± 0.0002 (*,^ 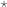 ^)	0.0332 ± 0.033 (**,^ 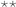 ^)	0.0027 ± 0.002 (**,^ 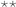 ^)	0.0369 ± 0.0031 (**,^ 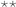 ^)
Cp	0.00335 ± 0.0002 (*,^ 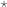 ^)	0.0390 ± 0.037 (**,^ 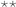 ^)	0.0028 ± 0.002 (**,^ 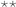 ^)	0.0448 ± 0.0043 (***,^ 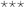 ^)
ES+Cp	0.00152 ± 0.0001 (***,^ 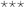 ^)	0.0227 ± 0.028 (*,^ 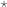 ^)	0.0012 ± 0.001 (**,^ 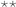 ^)	0.0262 ± 0.002 (**,^ 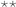 ^)
BS+Cp	0.00391 ± 0.0003 (ns,ns)	0.0427 ± 0.051 (***,^ 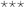 ^)	0.0032 ± 0.002 (*,^ 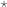 ^)	0.0500 ± 0.0071 (****,^ 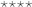 ^)

**Table 2:** Effect of soil amendment treatments on HQ (Hazardous Quotient) of Copper and Zinc from plant material of canola. Data were analyzed using One-Way ANOVA following Tukey multiple comparison test (*p* < 0.05). * showed statistical significance with respect to insecticide and ^
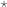
^ with respect to untreated control (ns: *p* > 0.05, *: *p* < 0.05, **: *p* < 0.01, ***: *p* < 0.001, ****: *p* < 0.0001, ^
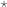
^: *p* < 0.05, ^
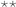
^: *p* < 0.01, ^
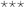
^: *p* < 0.001, ^
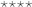
^: *p* < 0.0001).

Nature of Treatments	Field Experiments of 2019		Field Experiments of 2020	
	Copper	Zinc	Copper	Zinc
Insecticide	0.0145 ± 0.0013	0.0587 ± 0.0061	0.0056 ± 0.0003	0.0704 ± 0.0056
Control	0.0144 ± 0.0013 (ns)	0.0580 ± 0.0061 (ns)	0.0060 ± 0.0003 (ns)	0.0813 ± 0.0061 (ns)
ES	0.0110 ± 0.001 (*,^ 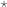 ^)	0.1356 ± 0.0237 (***,^ 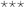 ^)	0.0022 ± 0.0001 (****,ns)	0.1532 ± 0.0371 (***,^ 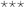 ^)
BS	0.0112 ± 0.001 (*,^ 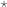 ^)	0.1107 ± 0.0211 (**,^ 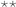 ^)	0.0027 ± 0.0001 (ns,^ 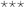 ^)	0.1229 ± 0.0274 (**,^ 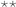 ^)
Cp	0.0112 ± 0.001 (*,^ 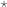 ^)	0.1299 ± 0.0178 (**,^ 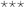 ^)	0.0028 ± 0.0001 (**,^ 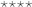 ^)	0.1494 ± 0.0281 (**,^ 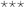 ^)
ES+Cp	0.0051 ± 0.0001 (***,^ 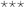 ^)	0.0758 ± 0.091 (*,^ 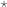 ^)	0.0012 ± 0.0001 (**,^ 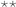 ^)	0.0872 ± 0.0047 (*,^ 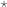 ^)
BS+Cp	0.0130 ± 0.0003 (ns,ns)	0.1425 ± 0.0061 (****,^ 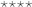 ^)	0.0032 ± 0.0001 (ns,^ 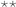 ^)	0.1667 ± 0.0411 (****,^ 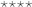 ^)

**Table 3:** Weather conditions during field experiments.

Crop Months	Temperature (°C)		Relative Humidity (%)		Rainfall (mm)	
	2018–19	2019–20	2018–19	2019–20	2018–19	2019–20
November	17.5	19.55	84	68.8	0.6	3
December	14.1	11.5	81.5	77	0.7	7
January	13.1	12.8	80.7	79	18	5
February	14.7	16.25	79	64.9	64.2	24.8
March	19.9	19.2	68.5	73.7	55.7	135

**Table 4:** Properties of field soil used in both year experiments.

Physical and Chemical Properties of Soil	Field Experiments	
	Year 2018–19	Year 2019–20
Sand (%)	51.1	51.5
Silt (%)	35.7	35.4
Clay (%)	14.7	15
pH	8.31	8.50
Saturation (%)	31.7	31.6
ECe (dSm^−1^)	1.5	1.5
Organic Matter (%)	0.57	0.61
Ca^2+^ + Mg^2+^ (mmol_c_/L)	12.51	13.54
Cu (Total) (mg kg^−1^)	24.5	24.4
Fe (Total) (mg kg^−1^)	121	124
Nitrogen (Total) (%)	0.047	0.045
Phosphorous (Available) (ppm)	7.50	7.63
Potassium (Extractable) (ppm)	145	155
Sulfur/SO_4_^2−^(ppm)	18.7	19.2
Zn (mg kg^−1^)	1.85	1.97

**Table 5:** Properties of bio-sulfur and compost.

Characters (Units)	Bio-Sulfur	Compost
Carbon (g kg^−1^)	---	210.5
Nitrogen (g kg^−1^)	25	17.1
Total Phsophorous (g kg^−1^)	15	3.01
Olsen Phsophorous (g kg^−1^)	295.5	259.3
Calcium (g kg^−1^)	0.05	0.053
Carbon:Nitrogen	---	17.2
Carbon:Phosphorous	---	72.5
pH	6.2	6.43
Sulfur (%)	70	5.8

**Table 6:** SAT under field conditions.

Sr. No.	Treatments	Specifications
1	T1	Insecticide (Positive Control)
2	T2	Untreated control
3	T3	Elemental Sulfur
4	T4	Bio-Sulfur
5	T5	Compost
6	T6	Elemental Sulfur + Compost
7	T7	Bio-Sulfur + Compost

## Data Availability

The data is contained within the manuscript. Further inquiries can be directed to the corresponding authors.
